# Validity of the Kinect for Gait Assessment: A Focused Review

**DOI:** 10.3390/s16020194

**Published:** 2016-02-04

**Authors:** Shmuel Springer, Galit Yogev Seligmann

**Affiliations:** 1 Department of Physical Therapy, Faculty of Health Sciences ,Ariel University, Kiryat Hamda, Ariel 40700, Israel; 2 Faculty of Medicine, Tel-Aviv University, Tel Aviv 69978, Israel; galit.yogev@gmail.com; 3 Functional Brain Center, Wohl Institute for Advanced Imaging, Tel-Aviv Sourasky Medical Center, Tel Aviv 69978, Israel

**Keywords:** Kinect, validity, gait analysis

## Abstract

Gait analysis may enhance clinical practice. However, its use is limited due to the need for expensive equipment which is not always available in clinical settings. Recent evidence suggests that Microsoft Kinect may provide a low cost gait analysis method. The purpose of this report is to critically evaluate the literature describing the concurrent validity of using the Kinect as a gait analysis instrument. An online search of PubMed, CINAHL, and ProQuest databases was performed. Included were studies in which walking was assessed with the Kinect and another gold standard device, and consisted of at least one numerical finding of spatiotemporal or kinematic measures. Our search identified 366 papers, from which 12 relevant studies were retrieved. The results demonstrate that the Kinect is valid only for some spatiotemporal gait parameters. Although the kinematic parameters measured by the Kinect followed the trend of the joint trajectories, they showed poor validity and large errors. In conclusion, the Kinect may have the potential to be used as a tool for measuring spatiotemporal aspects of gait, yet standardized methods should be established, and future examinations with both healthy subjects and clinical participants are required in order to integrate the Kinect as a clinical gait analysis tool.

## 1. Introduction

Restoration of independent and functional community ambulation is a major rehabilitation goal. A comprehensive gait analysis can detect deviations and impairments underlying reduced function, and thus may assist in clinical decision making as well as in quantifying rehabilitation effectiveness. Clinical gait analysis may also be a useful tool to distinguish between disease entities and to assess general health and risk of disease or injury such as fall detection and prediction among the elderly population [1,2,3].

Basic clinical gait assessments are mainly observational or based on gait speed, and are appropriate for evaluating and monitoring functional status and overall health in a wide range of populations [4]. Yet, they lack the precision and data richness of instrumented methods that provide the kinematic and spatiotemporal aspects of the gait cycle that are crucial for comprehensive gait analysis [2,3]. However, instrumented gait analysis requires expensive equipment which is not always available in clinical settings.

Recent evidence suggest that Microsoft Kinect, originally developed as a video gaming device to track the movements of a player interacting with a game, can be used for assessment of spatiotemporal gait variables [5] as well as gait kinematics [6]. The Kinect consists of an array of sensors, including a camera and a depth sensor, enabling the Kinect to track and record 3-D human motion without using controllers or markers. The system records live videos with a conventional camera and integrates these with depth information comprising a combined feed from emitted infrared light and an infrared camera. The Software Development Kit (SDK) then detects the human subject in the 3-D video in real-time and extracts an artificial skeleton with joints motion over time. As compared to traditional gait analysis systems, the Kinect’s price is very low, and so has the potential to be used as a low-cost alternative motion analysis tool. Nevertheless, the validity of this gait measurement tool has to be well established before it can be used in routine scenarios [7]. The purpose of this work is to identify and critically evaluate the literature describing the concurrent validity of using Kinect as an instrument for gait analysis. Specifically, we aimed to test the accuracy of the Kinect for assessment of various gait parameters as compared to traditional gait analysis systems. The current report was written in accordance with the Preferred Reporting Items for Systematic Reviews and Meta-Analysis (PRISMA) statement [8].

## 2. Methods

### 2.1. Search Strategy

Online searches of the PubMed, CINAHL, and ProQuest databases were performed. The search terms used were: “Kinect”, “Microsoft XBOX”, crossed with “Gait”, “Locomotion”, “Walking”, and “Ambulation“. The search was restricted to the English language. The last full search was conducted in October 2015. The search was firstly performed by the authors independently (Shmuel Springer and Galit Yogev Seligmann) and finalized by the authors in collaboration. Duplicate publications were deleted after all databases and reference lists were searched. The titles and abstracts of all relevant papers were reviewed, with the full article reviewed whenever considered necessary to reach a conclusion about inclusion.

### 2.2. Eligibility Criteria

The authors screened all selected citations independently. Study inclusion criteria were: (1) studies of human subjects in which gait analysis data was recorded and assessed with the Kinect as well as with another gold standard device; (2) trials appearing in refereed journals; and (3) a report of at least one numerical finding of spatiotemporal or kinematic measures assessing gait. Exclusion criteria were: studies reported in conference proceedings, posters, theses, or dissertations.

### 2.3. Data Extraction and Quality Assessment

The extracted study details focused on participant characteristics, study protocols, the data capture method from the Kinect, the gait analysis model, the outcome measures, the gold standard device used for validation, and the statistical methods.

The quality of study design and performance are key critical features in evaluating scientific data. Although a large body of literature exists to provide guidelines for the systematic evaluation of research methodology [8,9], the majority are focused primarily on studies of healthcare interventions; in particular, randomized controlled trials. As no standardized or recognized guidelines were found for reviews of validity, we used the quality appraisal form suggested by McGinley *et al.* [7]. This form was developed for a systematic review intended to assess the reliability of three-dimensional kinematic gait measurements. The appraisal component integrates relevant examples of methodological quality criteria from other systematic reviews, gait classification, quality criteria proposed for the measurement properties of health status questionnaires, and the Quality Assessment of Diagnostic Accuracy Studies QUADAS tool used to appraise studies of diagnostic accuracy [10,11,12]. Appraisal items were not given grade as the validity of an appropriate scoring systems is presently unverified [9]. The appraisal criteria included themes related to external validity such as sampling methods and participants’ characteristics, standardization and protocol description, and selection of statistical methods.

The data extraction and appraisal form were used independently by the authors (Shmuel Springer and Galit Yogev Seligmann) to extract key details from each report and to evaluate its quality. Rating differences on quality criteria were reassessed against the original paper to ascertain the correct evaluation.

## 3. Results

### 3.1. Study Selection

Initial screening by search terms yielded a total of 366 papers; 348 were considered irrelevant on the basis of their title and abstract. The 18 potentially relevant papers were retrieved in full text form for further evaluation. Four studies were excluded as no numerical findings of spatiotemporal or kinematic measures assessing gait were reported. Another two studies were excluded as no gold standard was used for validation of the data recorded by the Kinect system. Ultimately, 12 papers were included in the review. The literature search was conducted in October 2015.

### 3.2. Methodological Quality

The key criteria for quality indicators are reported in Table 1.

Sample selection: In the majority of the studies the sampling method for recruitment of gait participants was not stated. In the reports with healthy subjects, it is most probable that convenience sampling was used. In studies with clinical participants, the sampling method was also based on convenience sample from local clinics [13,14,15,16].

Inclusion/exclusion criteria and description of gait participants: Only five studies clearly stated the eligibility criteria for gait participants. Of these five studies, two included only healthy subjects [17,18], one included clinical participants and healthy controls [14], and two included only clinical participants [15,16]. The other studies that included only healthy subjects specified a limited criterion such as the absence of previous musculoskeletal disorders, or the subjects being injury-free individuals [5,19,20,21], and one study did not specify any criterion [22]. Behrens *et al.* [13] tested patients with multiple sclerosis (MS) and healthy controls, and mentioned the method with which the MS patients were diagnosed, while no criteria for healthy controls were included. The quality of the descriptions of the gait participants also varied across the reports. In only nine studies gait participants were sufficiently described with regard to age, gender, health status, and anthropometric characteristics [5,15,16,17,18,19,20,21,23].

Procedure, model description, and outcome measures: All the reports included a detailed description of the study procedure and outcome measures. However, in some studies, detailed specification of the Kinect-participant distance was missing from the experiment setup description [5,16,18,19]. The data capture method from the Kinect was generally adequately described with all reports providing adequate overall descriptions of the models used, or providing appropriate reference to available descriptions.

Statistical analysis: Bland-Altman analysis (used to examine the agreement between two different measurement techniques) was used in seven of the 12 studies [5,13,14,18,19,21,23]. Concordance correlation coefficients (*r_c_*) were also computed in some reports [5,19,23], as well as linear regression analyses to calculate the slope of the relationship between Kinect and the gold standard [5,18]. One study presented basic descriptive statistics (e.g., mean, standard deviation) of gait cycle detection errors of the Kinect and a gold standard system without presenting the agreement between the two different measurements [22], while other studies used correlation coefficients [15,16,17,20] or analyzed consistency using intraclass correlation coefficient (ICC) [21] to identify agreement with the gold standard.

**Table 1:** Methodological quality of the reviewed articles.

Study	Gait Participants			Protocol Description	Model Description	Outcome Description	Statistical Methods
	Sampling Method	Eligibility Criteria	Description				
Auvinet *et al.* [22]	Not stated	Not stated	Partial	Adequate	Adequate	Adequate	Limited
Auvinet *et al.* [20]	Not stated	Limited	Adequate	Adequate	Adequate	Adequate	Limited
Clark *et al.* [5]	Not stated	Limited	Adequate	Partial	Adequate	Adequate	Adequate
Galna *et al.* [14]	Convenience	Stated	Partial	Adequate	Adequate	Adequate	Adequate
Behrens *et al.* [13]	Convenience	Limited	Partial	Adequate	Adequate	Adequate	Adequate
Mentiplay *et al.* [23]	Not stated	Limited	Adequate	Adequate	Adequate	Adequate	Adequate
Pfister *et al.* [18]	Not stated	Stated	Adequate	Partial	Adequate	Adequate	Adequate
Xu *et al.* [19]	Not stated	Limited	Adequate	Partial	Adequate	Adequate	Adequate
Paolini *et al.* [17]	Not stated	Stated	Adequate	Adequate	Adequate	Adequate	Limited
Geerse *et al.* [21]	Not stated	Limited	Adequate	Adequate	Adequate	Adequate	Adequate
Clark *et al.* [15]	Not stated	Stated	Adequate	Adequate	Adequate	Adequate	Adequate
Vernon *et al.* [16]	Not stated	Stated	Adequate	Partial	Adequate	Adequate	Adequate

**Table 2:** Characteristics of the identified studies.

Study	Participant Characteristics (Age, Type, Gender)	Outcome Measures	a. Kinect Version b. Number of Sensors. c. Orientation & Distance	Type of Data (*i.e.*, Skeletal Data, RGB Data, or Raw Depth Data)	Type of Gold Standard	Main Findings
Auvinet *et al.* [22]	*n* = 11 YA Age: 24.6 ± 3.2 years Gender-NA	Heel-strike detection error; stride duration	a. V1 b. One sensor c. 2 m in front of the subject	Depth data	120 Hz Vicon system 3D motion analysis	Heel strike errors were somewhat higher for the Kinect compared to the gold standard, mean cycle duration error were almost similar in both systems.
Auvinet *et al.* [20]	*n* = 15 YA Age: 25.3 ± 3.6 years Gender: M-12, F-3	Traditional *vs.* New asymmetry index	a. V1 b. One sensor c. 2 m in front of the subject	Depth data	120 Hz Vicon system 3D motion analysis	The new proposed index distinguished asymmetrical gait using the Kinect while traditional model did not. High correlation was found for the asymmetry computed by the Kinect using the new method and the gold standard.
Clark *et al.* [5]	*n* = 21, YA Age: 26.9 ± 4.5 years, Gender: M-10, F-11	Step time; Step length; Gait speed; Stride time; Stride length; Foot swing velocity	a. V1 b. One sensor c. In front of the participant (distance not available)	Skeletal data	120 Hz Vicon 3D motion analysis	Gait speed, step length and stride length possessed excellent overall agreement with gold standard, while other parameters possessed only modest to poor overall agreement.
Galna *et al.* [14]	*n* = 9 PWPD Age 68.2 ± 8.3 years Gender: M-3, F-6 *n* = 10 YA Age 27.5 ± 5 years Gender: M-5, F-5,	Vertical displacement of the knee during walking on spot: Timing of movement and spatial displacement	a. V1 b. One sensor c. 3 m in front of the subject	Skeletal data	100 Hz Vicon 3D motion analysis	In comparison to the gold standard, timing of movement repetitions measured by the Kinect was very accurate. However, the Kinect had limited success measuring spatial vertical displacement.
Behrens *et al.* [13]	*n* = 22 PWMS Age 43 ± 9 years, Gender: M-9, F-13 YA *n* = 22 Age 37 ± 11 years Gender: F-13, M-9	Gait speed	a. V1 b. One sensor c. 2 m in front of the subject	Skeletal data	Gait speed measured by the Timed 25-Foot Walk test	Moderate correlation was found between average gait speed measured with the Kinect and the clinical measure.
Mentiplay *et al.* [23]	*n* = 30 YA Age: 22.87 ± 5.08 years, Gender: M-15, F-15	Gait peed; speed variability; step length; step width and time; foot swing velocity; medial–lateral and vertical pelvis displacement. Kinematic outcome measures: ankle flexion; knee flexion and adduction; hip flexion.	a. V2 b. One sensor c. 8 m in front of the subject	Skeletal data	100 Hz Vicon 3D motion analysis	Excellent overall agreement with the gold standard was shown for gait speed and step time only.
Pfister *et al.* [18]	*n* = 20 YA Age: 27.4 ± 10.0 years. Gender: M-9, F-11	Maximum angular displacement for hip and knee flexion and extension; Stride timing.	a. V1 b. One sensor c. To the subject’s left at a 45° to treadmill (distance not available)	Skeletal data	120 Hz Vicon 3D motion analysis	Kinect and gold standard hip angular displacement correlation was very low and error was large. Kinect knee measurements were somewhat better than hip, but were not consistent enough for clinical assessment. Stride time correlation was high and error was fairly small.
Xu *et al.* [19]	*n* = 20 YA Age: 28.5 ± 8.2 years Gender: M-10, F-10	Step time; stride time; swing time; stance time; double limb support time. Kinematic outcome measures: hip and knee joint angles over a gait cycle.	a. V1 b. One sensor c. In front of treadmill (distance not available)	Skeletal data	60 Hz Optotrak System 3D motion analysis	Step time, stride time, and step width showed excellent overall agreement with gold standard. Kinematic parameters showed poor overall agreement.
Paolini *et al.* [17]	*n* = 12 YA Age: 32 ± 5 years Gender M-7, F-5	Mean values of a 3D foot position over the trial duration, and root mean square deviation (RMSD).	a. V1 b. One sensor c. 1 meter in front of treadmill	RGB data	50 Hz Vicon 3D motion analysis	Foot position error and deviations were small compared to gold standard.
Geerse *et al.* [21]	*n* = 21 YA Age: 30.2 years Gender: M-11, F-10	Raw data of body point’s time series, and spatiotemporal gait parameters: gait speed, cadence, step length, stride length, step width, step time, stride time.	a. V2 b. Four sensors c. 0.5 m from the left border of the walkway with an angle of 70°. The first sensor was positioned at 4 m from the start point, other 3 sensors were placed at inter distance of 2.5 m	Skeletal data	60 Hz Optotrak System 3D motion analysis, 10 MWT time	Good to excellent agreement with gold standard for raw data and all spatiotemporal gait parameters.
Clark *et al.* [15]	*n* = 30 PPS Age 68 ± 15 years, Gender: M-21, F-9	Step length and gait speed	a. V1 b. One sensor c. Patients walked towards the Kinect camera, stopping 0.5 m in front of it	Skeletal data	10MWT time and number of steps	Good correlation was found between gait speed, and step length measured with the Kinect and the clinical measures.
Vernon *et al.* [16]	*n* = 30 PPS Age 68 ± 15 years, Gender: M-21, F-9		a. V1 b. One sensor c. Off-center from the starting point of the TUG test (distance not available)	Skeletal data	TUG clinical test	TUG time measured by stopwatch and Kinect showed excellent association

YA = Young Adults; PWPD = Patients with Parkinson’s disease; PWMS = Patients with MS; PPS—Patients post stroke; 10MWT—10-m walking test; TUG—Timed Up and Go.

### 3.3. Characteristics of the Included Studies

Table 2 summarizes some of the salient features of these investigations.

Subjects: The 12 studies reviewed included 273 overall participants: 182 participants were healthy adults (ages ranged from 19 to 63 years), one study included 22 patients with MS (43 ± 9 years) [13], two studies included 30 patients post stroke (68 ± 15 years), and one study included nine patients with Parkinson’s disease (68.2 ± 8.3 years) [14]. The number of subjects per group ranged from 9 to 30 (mean 19.50 ± 7.32).

Synchronization of data between the gold standard and the Kinect: Out of the 12 studies, nine studies used a 3D motion analysis to validate the data collected by the Kinect system. Specifically, seven studies used the Vicon system (Vicon, Oxford, UK), and two studies used the Optotrak Certus system [19,21]. While these systems’ sampling rates ranged between 60 to 120 Hz, the Kinect sampling rate was 30 Hz. Therefore, in these studies spline interpolation was used to resample the Kinect data to the frequency of sampling by the gold standard system. Four studies [13,15,16,21] compared data measured by Kinect to clinical test measured by a stop watch and number of steps, such as the Timed 25-Foot Walk test [13], the 10-m walking test [15,21] , and the Timed Up and Go test [16].

Calculating gait cycle events from Kinect: Most studies used the events and variables derived from the anatomical landmark data provided by the Microsoft Kinect skeleton tracking algorithm. Two studies used the raw depth data provided by the Kinect [20,22], and one study used the RGB data for the tracking procedure [17]. The method used to detect gait cycle events varied among the studies. For example, two studies used gait event time points of toe-off and ground contact to identify phases of the gait cycle [5,23]. The studies conducted by Auvinet et al. [20,22] estimated heel strike events indirectly by searching for the extreme values of the distance between the knee joints. Another study used the time from peak hip/knee flexion to peak hip/knee flexion of the same limb in order to define stride timing [18]. As can be seen in Table 2, the orientation of the Kinect system also differ between the studies, with some studies even not mentioning the exact distance relative to participant [5,18,19]. In addition, only one study used multiple Kinect set-ups to increase the measurement volume [21].

### 3.4. Validity Findings

Pearson’s correlation coefficient assesses precision (relative agreement, *r*) while concordance coefficient evaluates both precision and deviations from the line of identity (over all agreement, *r_c_*) [5]. We reported on the Pearson’s correlation or ICC (consistency), and concordance coefficients unless one of them was not available. Correlations were interpreted according to the following scale: poor (<0.40), modest (0.40–0.74), or excellent (>0.75) [24].

Spatiotemporal measures: Eight studies validated spatiotemporal variables [5,13,15,16,18,19,21,23]. In all eight studies, agreement between Kinect and the gold standard was assessed using Bland-Altman 95% bias and limits of agreement (LoA), Pearson’s correlation coefficients, ICC, concordance correlation coefficients, or at least one of these tests.

Table 3 summarizes the relative and overall agreement of all spatiotemporal parameters measured in these studies. The following parameters showed relative and/or overall agreement in at least one study. Gait speed was assessed in six studies: in five studies [5,15,16,21,23] the relative and overall agreement were excellent while the third study [13] showed only a moderate relative agreement. Step time and stride time were assessed in four studies each [5,18,19,21,23]. Relative agreement ranged from moderate to excellent. The overall agreement ranged from poor to excellent (see Table 3). Foot swing velocity was assessed in two studies [5,23] and showed excellent relative agreement but moderate to poor overall agreement.

Step width, step length, and stride length, showed all excellent relative agreement. Over all agreement ranged from poor to excellent for both step width and step length, and showed excellent agreement for stride length.

Auvinet *et al.* [20] proposed a new asymmetry index using Kinect which is based on the longitudinal spatial dissimilarities between lower-limb motions during the gait cycle. The correlation between the asymmetry with the proposed method and asymmetry measured by a gold standard motion capture data was of 0.968, while indices based on spatiotemporal gait parameters of the Kinect skeleton failed to recognize asymmetric gait.

Kinematic measures: Table 4 summarizes the relative and overall agreement of kinematic parameters measured in five studies. Concordance correlation coefficients or ICC, was available only in three studies [19,21,23], while the other two studies reported error values [18] or limits of agreement of 95% [14]. In general, the studies demonstrated varied results. While some parameters exhibit excellent between-systems agreement, other kinematic parameters show low overall agreement and large error, which are not consistent enough for clinical assessment.

## 4. Discussion

The purpose of this review was to summarize the cumulating evidence referring to the validity of gait assessment taken by Kinect in comparison to a gold standard. As far as we know, this is the first time that validation of the Kinect system is reviewed.

Although only a few studies explored a full comprehensive assessment [18,19,21,23], findings consistently showed superiority for the validity of spatiotemporal parameters compared to kinematic parameters. These studies concluded that while Kinect may not accurately record body kinematic data, it shows good potential as a tool for measuring some spatiotemporal parameters of gait.

In a clinical perspective, factors that militate against the use of gait analysis tool in clinical settings are lack of availability, reimbursement, and training [2]. The Kinect, a device that is low-cost, widely available, does not require training, and is free of markers or sensors attached to the body, has the potential to improve the feasibility of clinical gait analysis. Nonetheless, more research is required to determine the validity of the Kinect for a wider variety of spatiotemporal parameters. Furthermore, improvements in software and hardware are essential to enhance Kinect sensitivity for kinematic measures.

The reviewed studies varied in methodology as to capturing gait data and testing conditions. For example, Auvinet *et al.* [20,22] estimated heel strike events indirectly by searching for the extreme values of the distance between the knee joints, while Mentiplay *et al.* [23] used event time points of toe off and ground contact to identify phases of the gait cycle. Furthermore, while some gait studies were based on over-ground walking [5,13,14,15,16,21,23], a number of other studies were based on treadmill walking [17,18,19,20,22]. The positions of the Kinect sensor also differ between the studies. For example, Pfister *et al.* [18] positioned the Kinect sensor to the subject’s left at a 45° angle to the treadmill, while Xu *et al.* [19] placed the Kinect sensor in front of the treadmill. The accuracy of the measurements given by the Kinect is dramatically affected by the sensor position and tracking methodology [25]. All of the above variations may explain some of the difference in validity between the reports. Consequently, a custom and standardized methodological procedure for examining gait using the Microsoft Kinect sensor is required before it can be implemented in the clinical setting.

A number of limitations should be considered when interpreting the findings of this report. This review attempted to be comprehensive, yet there is a probability that some relevant articles were missed. It is possible that some articles were not captured by search keywords or went unreported due to publication forms not included in our criteria, such as studies published in conference proceedings. In addition, most subjects in the reviewed papers were young and healthy, and the studies that tested clinical participants were mainly focused in testing the Kinect reliability (e.g., test-retest), while the validity was tested against clinical assessment and not against instrumented tools, gold standards which are considered more valid. Therefore, the ability of the Kinect to comprehensively assess abnormal clinical gait patterns in clinical settings was not fully validated.

**Table 3:** Correlation coefficient (*r*), Concordance correlation coefficients (*r_c_*), and Intraclass correlation coefficient (ICC) of gait parameters measured by Kinect and gold standard (Spatiotemporal Parameters).

Outcome Measure	Clark *et al.* [5]	Behrens *et al.* [13]	Pfister *et al.* [16]	Mentiplay *et al.* [23] *	Xu *et al.* [19]	Geerse *et al.* [21] *	Clark *et al.* [15] ****	Vernon *et al.* [16]
Gait speed	*r* = 0.95 ***r_c_* = 0.93**	*r* = 0.44		*r* = 0.99 ***r_c_* = 0.90**		ICC = 0.99	*r* = 0.92	*r* = 0.99
Step time	*r* = 0.82 ***r_c_* = 0.23**			*r* = 0.92 ***r_c_* = 0.75**	*r* = 0.77 ***r_c_* = 0.75**	ICC = 0.89		
Stance time	-				*r* = 0.57 ***r_c_* = 0.37**			
Stride time	*r* = 0.69 ***r_c_* = 0.14**		*r* > 0.80		*r* = 0.92 ***r_c_* = 0.92**	ICC = 0.96		
double limb support time	-				*r* = 0.24 ***r_c_* = 0.10**			
Foot swing velocity	*r* = 0.93 ***r_c_* = 0.54**			*r* = 0.79 ***r_c_* = 0.11**	*r* = 0.43 ***r_c_* = 0.21**			
Speed variability	-			*r* = 0.75 ***r_c_* =** **0.0**				
step width	-			*r* = 0.94 ***r_c_* =** **0.0**	*r* = 0.82 ***r_c_* =** 0.82	ICC = 0.65		
medial-lateral and vertical pelvis displacement	-			*r* = 0.45 ***r_c_* =** **0.0**				
Step length	*r* = 0.99 ***r_c_* = 0.97**			*r* = 0.90 ***r_c_* = 0.13**		ICC = 0.99	*r* = 0.86	
Stride length	*r* = 0.99 ***r_c_* = 0.99**					ICC = 0.99		
Cadence						ICC = 0.97		

* Reported values refer to comfortable walking pace, **** Reported values refer to manually assessed time and affected limb.

**Table 4:** Correlation coefficient (*r*), Concordance correlation coefficients (*r_c_*), and Intraclass correlation coefficient (ICC) of gait parameters measured by Kinect and gold standard. (Kinematic Parameters).

Outcome Measure	Pfister *et al.* [18] **	Geerse *et al.* [21]	Xu *et al.* [19]	Mentiplay *et al.* [23] *	Galna *et al.* [14] ***
Vertical displacement of the knee during walking on spot					HC *r* = 0.822 (LoA_95%_ = 66.80) PWPD *r* = 0.848 (LoA_95%_ = 123.37)
Nineteen matched body points in AP, ML and V directions		ICC was generally >0.60 for all directions; yet, some time series demonstrated poor to fair agreement e.g., Left Ankle: AP ICC = 0.970 ML ICC = 0.871 V ICC = 0.392			
Ankle flexion				*r* = 0.11 ***r_c_* = 0.01**	
Peak knee flexion-swing	Left knee *r* = 0.79 (−14.1 ± 7.05) Right knee *r* = 0.87 (−16.73 ± 5.45)			*r* = −0.05 ***r_c_* = −0.02**	
Peak knee flexion contact				*r* = −0.01 ***r_c_* =** −**0.01**	
Knee adduction				*r* = −0.07 ***r_c_* = 0.0**	
Knee extension	Left knee *r* = 0.78 (3.07 ± 6.11) Right knee *r* = 0.84 (4.43 ± 6.25)		*r* = 0.81 ***r_c_* = 0.41**		
Hip flexion	Left hip *r* = −0.06 (−10.81 ± 9.95) Right hip *r* = 0.15 (−8.12 ± 10.49)		*r* = 0.95 ***r_c_* = 0.71**	*r* = 0.49 ***r_c_* = 0.08**	
Hip extension	Left hip *r* = −0.22 (−2.55 ± 10.89) Right hip *r* = −0.32 (−7.84 ± 11.47)				

* Reported values refer to comfortable walking pace; ** Values in parentheses refer to error magnitude since *r_c_* was not stated; *** Values in parentheses refer to Limit of agreement magnitude since *r_c_* was not stated; HC = Healthy Controls; PWPD = Patients with Parkinson’s disease; AP = anterior-posterior; ML = mediolateral; V = vertical.

Another limitation is that apart from two studies [21,23] which stated the use with the newer version of the Kinect known as the Xbox One Kinect or Kinect V2, it is probable that all other studies used the first generation of the Kinect sensor (Xbox 360). As with the original Kinect, the Kinect V2 utilizes infrared to read its environment, yet it consists of features which improve its motion tracking capture capabilities over its predecessor. The Kinect V2 uses time of flight technology; it has an increased field of view, and a better resolution of the depth camera. These new features may potentially enhance the utility of this device for examining gait. Further investigations should be undertaken with the Kinect V2 and clinical participants.

Finally, apart from Geerse *et al.* [21] that evaluated multi Kinect set up, all the included studies conducted the validation using sing a single Kinect sensor, thus, having a limited measurement capacity. It is important that future research will examine ways to utilize Kinect in a clinical setting to ensure translation into standard clinical practice. Such studies should also evaluate how multiple set-ups of Kinect sensors affect the accuracy of the gait parameters. The promising results of the present review suggest that such studies are warranted.

## 5. Conclusions

The present review of 12 studies that assessed gait analysis with Kinect and a gold standard indicated good validity for only some spatiotemporal gait parameters, and poor validity for gait kinematics variables. The studies vary greatly in terms of their methodological capability for capturing gait data. In addition, most studies tested healthy subjects in laboratory settings. Thus, customization and standardization of methodological procedure for examining gait using the Kinect sensor, and further research involving people with gait pathologies, is required before it can be fully implemented for clinical use.
